# Filial piety and fertility decisions, Thailand

**DOI:** 10.2471/BLT.25.294174

**Published:** 2025-12-11

**Authors:** Nopphol Witvorapong, Jim Stankovich, Thant Zin

**Affiliations:** a Center of Excellence for Health Economics, Faculty of Economics, Chulalongkorn University, 254 PhayaThai Road, Pathumwan, Bangkok, 10330 Thailand.; b Faculty of Economics, Chulalongkorn University, Bangkok, Thailand.

Thailand is undergoing a drastic demographic transition, with rapid population decline and ageing. The total fertility rate fell from 6.0 births per woman in the 1960s to 1.2 in 2023, while life expectancy increased from 50.6 to 76.4 years in the past six decades. As a result, the World Bank estimates that, between 1960 and 2024, the youth dependency ratio dropped from 83.6% to 21.1%, while the old-age dependency ratio rose from 5.3% to 21.9%.^1^ A report forecasts that by 2040, the country will have the highest share of people aged 65 and older among countries in the World Bank’s East Asia and Pacific region.^2^

Population decline and ageing pose major challenges. Under the current economic structure, Thailand’s economic growth heavily depends on the size of its workforce, and is therefore negatively affected by population decline, as it leads to a labour supply shortage.^3^^,^^4^ The reliance on labour-intensive sectors is because the country has limited technological expertise,^3^ and quality of its workforce has shown minimal improvement over the past three decades, with over one third of workers currently having only primary education or lower.^4^

Population ageing also has an effect at the household level. Intergenerational relationships in Thailand are characterized by filial piety, defined as the sense of responsibility that children have towards older parents.^5^ Using data from the nationally representative 2024 Survey of Older Persons, we calculated population-weighted percentages from a sample of 39 173 individuals aged 50 years and older. Our findings showed that children represent an important source of old-age security. Financially, older parents tended to rely on themselves (58.9%) or their children (28.8%), while non-parents relied on themselves (74.4%) and other family members (12.6%). For older people experiencing functional limitations and needing care, children constituted the main source of care for their parents (68.1%), while it was other family members for non-parents (66.4%). In general, needs for filial support increase when older parents live in rural areas, have fewer children and possess no financial wealth. Unless the government takes a larger portion of the caregiving burden or older people are empowered to become more self-reliant, the shrinking family size will negatively affect the well-being of Thai older adults and how they are cared for.^6^

Adult children are similarly affected. Caring for older parents, with few siblings to share the responsibilities with, can cause burnout and depression.^7^^,^^8^ Furthermore, the opportunity cost associated with exiting the labour market because of caregiving is non-negligible. According to our findings from the 2024 survey, 10.8% of the 1166 respondents who cared for their parents had to quit their jobs. Although research on individuals who have to care for their children and their parents is limited for Thailand, in other countries, the negative impact is expected to be particularly severe. This generation of caregivers is at a higher risk of physical, psychological, economic and social problems.^7^^,^^8^

Counteracting the effects of population decline and ageing calls for a deeper understanding of fertility decisions and how to influence them. However, studies on this subject in Thailand remain scarce, as they mainly focus on implications of fertility decline^9^ or fertility needs of population subgroups.^10^ Studies identifying fertility causes explore interrelated factors that directly influence childbearing decisions, changing the costs and benefits of children. Such factors include personal characteristics (which determine households’ childrearing capabilities), fertility preferences and public policies (which affect both preferences and resources available to households).^11^

The role of social values in fertility decisions remains underexplored. Social values affect fertility outcomes through at least two channels. First, social values influence fertility preferences, impacting how individuals position themselves among their social groups. Studies have shown that culture and religious norms^12^ are linked to the desired number of children and contraceptive intentions. Second, values shape the expectations that households have regarding the experiences of being a parent and alter the expected costs and benefits of having children. Studies suggest that fertility decisions are partially driven by views regarding the division of labour between husbands and wives, and the adequacy of the State’s infrastructure (legal frameworks, parental leave legislation and flexible working arrangements, among others) that allows women to reconcile work with family affairs.^11^^–^^13^

In *The second national reproductive health development policy and strategy (2017–2026): promotion of quality birth and growth*, the Thai government explicitly acknowledges the role of values.^14^ The strategy suggests that reasons for younger generations choosing not to have children include: (i) placing higher priorities on career and financial aspirations, compared to family-building; (ii) feeling that having children would involve too much personal sacrifice; and (iii) having concerns over well-being of their children, particularly the perception that they would grow up in a challenging society. 

To better understand fertility decisions, we suggest that filial piety be examined, as it is an important value in Thai society. On the one hand, filial piety may promote fertility. Households that expect not to be completely self-reliant in old age, reside in countries where government support is limited, and believe, due to norms, that children will care for them (as is the case for Thailand)^5^^,^^6^ should find that having children is in their best interest. Moreover, since filial support is normally provided in a setting of intergenerational co-residence and it is common for co-resident grandparents to care for grandchildren,^15^ households may expect filial support in exchange for grandparental care for as long as the grandparents are sufficiently healthy. In this case, filial piety indirectly reduces the expected costs of childrearing, positively influencing fertility outcomes.

On the other hand, filial piety may discourage fertility. Governed by norms, people of reproductive age expect to serve as caregivers for older parents, which constrains the resources needed to have children. The negative effect on fertility is likely to be considerable if individuals expect to provide care for a long time and do not have siblings to share the responsibility with,^16^ as is the case for Thailand, with declining fertility rates and increasing life expectancy.^3^

The effect of filial piety on fertility remains an open question. Few studies empirically investigate the effect of parental caregiving on fertility. For example, a recent study using data from Australia^17^ found that fertility expectations decreased when individuals took on the caregiving responsibility of their parents, and the effect became larger as caregiving continued. While the study considered the effect of parental caregiving as it occurred, we hypothesize that fertility is affected not just by the contemporaneous act of parental caregiving but also by expectations for future parental caregiving, motivated by filial norms.

To investigate the effect of filial piety on fertility decisions, a specific household-level data set is required. Respondents should consist of generations of household members. The data set would ideally be nationally representative and longitudinal,^17^ tracking changes regarding fertility-related needs, expectations and decisions over time. Data should be rich enough to capture the entire ecosystem of fertility decisions. In particular, it should include individual-level information on family-related and fertility-related attitudes (including filial piety); parental caregiving needs and expectations; childcare needs; the quantity and quality dimensions of fertility intentions and outcomes; receipt of and expectations for government support,; career aspirations; and demographic characteristics.

Different study designs using quantitative data could be explored. Outcome variables may include the ideal number of children, the actual number of children, the discrepancy between the two quantities and the probabilities of having a first, a second or a third child. The main explanatory variables should include the extent to which parental caregiving is provided, the extent to which individuals feel obligated to care for older parents, and the level of filial support expectations regarding own parents in the future. Many empirical strategies are available. Examples include multivariable mixed regression models with fixed and random effects, adjusting for household and personal characteristics, and generalized difference-in-differences models,^17^ capturing how fertility decisions are affected by changes in parental caregiving needs and expectations within the same households and across different households over time.

However, such data are not available in Thailand. In particular, the Survey of Older Persons, which contains information that is closest to what is needed to test the hypothesis, is not adequate. The survey does not contain information on filial norms, fertility intentions nor parental caregiving behaviours at the individual adult-child level; it is also cross-sectional, making it difficult to establish causality between expectations of having to provide filial support and fertility choice.

Knowledge of how filial piety affects fertility decisions has an implication on the strategic focus of fertility-promotion policies. If the effect of filial piety is positive, then the government should strengthen filial norms (for example through education programmes) and incentivize intergenerational co-residence to promote grandparental care, which in turn reduces the costs of having children for working-age individuals and consequently encourages fertility. If the effect of filial piety is negative, as implied in studies conducted in other countries,^16^^,^^17^ then the government should focus on ensuring that older individuals can be financially and physically independent, thereby relieving the burden and freeing up resources needed for adult children to make positive fertility decisions. More specifically, the government could set up a system of individual retirement savings accounts where interest income is tax-exempt, and provide additional assistance for informal workers and rural residents, who normally do not have a pension. The government could also promote healthy lifestyles among older people and expand the supply of community-based or market-based long-term care services,^5^^,^^6^^,^^9^ ensuring that older people are functionally independent for as long as possible and that options for care that do not directly require adult children are available.
